# Early Versus Delayed Bidirectional Superior Cavopulmonary Anastomosis (BSCA) in Infants: A Comparison of Outcomes

**DOI:** 10.7759/cureus.97665

**Published:** 2025-11-24

**Authors:** Sachin Talwar, Balaji Chandhirasekar, Sanjeev Kumar, Sivasubramanian Ramakrishnan, Vishal V Bhende

**Affiliations:** 1 Cardiothoracic and Vascular Surgery, All India Institute of Medical Sciences, New Delhi, New Delhi, IND; 2 Cardiothoracic and Vascular Surgery, Sri Jayadeva Institute of Cardiovascular Sciences and Research, Bengaluru, IND; 3 Cardiovascular Radiology and Endovascular Interventions, All India Institute of Medical Sciences, New Delhi, New Delhi, IND; 4 Cardiology, All India Institute of Medical Sciences, New Delhi, New Delhi, IND; 5 Pediatric Cardiac Surgery, Bhanubhai and Madhuben Patel Cardiac Centre, Shree Krishna Hospital, Bhaikaka University, Karamsad, IND; 6 Pediatric Cardiac Surgery, Sri Sathya Sai Sanjeevani Centre for Child Heart Care and Training in Pediatric Cardiac Skills, Navi Mumbai, IND

**Keywords:** bidirectional superior cavopulmonary anastomosis, cyanotic congenital heart disease, fontan operation, single ventricle palliation, univentricular heart

## Abstract

Background and aims

Functionally univentricular hearts (UVH) are a relatively rare congenital heart defect and often require staged palliation leading to the Fontan circulation. The bidirectional superior cavopulmonary anastomosis (BSCA) or bidirectional Glenn shunt (BDG) is a key intermediate procedure that partially separates systemic and pulmonary circulations. Although traditionally delayed until six months of age, earlier BDG has become common. This study compared outcomes in patients undergoing BDG at six months or younger versus seven months to one year and examined time to Fontan completion (FO), outcome predictors, and the influence of early BDG in the Indian context.

Methods

In this retrospective observational study, 173 patients with UVH underwent BDG at age one year or younger from January 2011 to December 2020 at a single tertiary care center. Twenty-seven patients (Group A) were six months or younger, and 146 (Group B) were seven to 12 months. Preoperative, intraoperative, and postoperative data were collected, including morbidity, mortality, and follow-up assessments. The data were statistically analyzed by Fisher’s exact test, Pearson’s chi-square test, and Cox proportional hazards or logistic regression modeling.

Results

Early mortality was 6.93% (12/173). Mortality was significantly higher in Group A (5/27; 18.52%) than in Group B (7/146; 4.79%; p=0.01). On multivariate analysis, preoperative mechanical ventilation, maximum inotropic score, and maximum vasoactive inotropic score (VIS) were independent predictors of mortality rather than patient age alone. Despite the higher initial risk, infants in Group A benefited from earlier unloading of the systemic ventricle and demonstrated less pulmonary artery (PA) distortion at follow-up.

Conclusions

Although BDG before age six months carries a higher early mortality risk, primarily linked to clinical severity rather than age, it can offer important advantages in systemic ventricle offloading and reduced PA distortion over time. These findings underscore the need for careful patient selection and highlight the potential for improved long-term outcomes with early BDG in appropriately selected infants.

## Introduction

Functionally univentricular hearts (UVH) comprise up to 10% of all congenital heart defects. The natural progression of UVH often includes reduced pulmonary blood flow, systemic desaturation, diminished quality of life, and early mortality. However, some patients with balanced systemic and pulmonary circulation may grow up without requiring surgery [1].

These patients typically undergo several palliative procedures early in life. One of the key steps in staged palliation is the bidirectional superior cavopulmonary anastomosis (BSCA) or bidirectional Glenn shunt (BDG). This procedure involves an end-to-side anastomosis of the superior vena cava (SVC) to the ipsilateral pulmonary artery (PA), thereby partially separating systemic and pulmonary circulations [2].

The BDG increases systemic oxygen saturation by offloading the systemic ventricle. BDG may be performed alongside additional procedures, such as PA banding, PA reconstruction, atrioventricular valve repair, and correction of anomalous systemic or pulmonary venous return [3]. The ultimate goal is to establish Fontan circulation, which improves systemic oxygenation, balances pulmonary and systemic blood flow, and enhances quality of life.

Although originally conceived as the definitive palliative surgery for patients with UVH physiology, the BDG has evolved into an intermediate-stage procedure for high-risk patients for primary Fontan completion (FO) [4]. Surgical outcomes following staged palliation have demonstrated significant improvements in survival and reduced morbidity, prompting its routine use at many centers before FO [5].

Traditionally, the BDG was deferred until after six months of age due to concerns about variable pulmonary vascular resistance and the potential for suboptimal outcomes in younger patients [6-8]. However, advancements in understanding hemodynamics, availability of pulmonary vasodilators, refined surgical techniques using microvascular clamps and monofilament sutures, and improved patient selection have made earlier BDG more feasible [9,10]. PA abnormalities can also be addressed during surgery in smaller infants. Early BDG offers an opportunity to correct PA distortions, perform PA plasty, and repair associated anomalies such as atrioventricular valve regurgitation and anomalous venous return, which, if unaddressed, can adversely affect systemic and pulmonary circulation.

As a result, BDG is now increasingly performed in infants younger than six months in Western centers, with growing evidence supporting its safety and efficacy. However, data from India on early and delayed outcomes in this age group remain limited. Therefore, we aimed to comparatively analyze the outcomes of patients undergoing BDG at the age of six months or younger versus those aged seven to twelve months at a single tertiary care center over ten years. We evaluated the interval to FO and examined the influence of early surgery timing and outcome predictors in the Indian context, contrasting these findings with available data from Western literature. The primary objective of this study was to compare early and delayed BDG (≤ 6 months versus seven to 12 months) with respect to early mortality and perioperative outcomes. Secondary objectives included assessing predictors of early mortality, evaluating postoperative morbidity and follow-up outcomes, and analyzing the interval to FO within the Indian clinical context. An exploratory objective was to examine factors influencing long-term Fontan readiness.

## Materials and methods

In this retrospectively designed observational study, we enrolled patients with UVH who were subjected to BDG at the age of one or younger between January 2011 and December 2020 at the Department of Cardiothoracic and Vascular Surgery, All India Institute of Medical Sciences, New Delhi. The research received approval from the Institutional Research Ethics Committee (Ref. No. IECPG-181/24.02.2021, OT-09/28.08.2025, dated 29.08.2025).

Inclusion and exclusion criteria

The study population comprised 27 patients aged six months or younger (Group A) and 146 patients aged seven months to one year (Group B). Emergency BDG cases were excluded.

Data collection

The baseline data, representing age, sex, weight, height, and body surface area, were obtained using a structured proforma. Clinical history, symptom duration, and preoperative oxygen saturation were noted. Preoperative investigations included hemoglobin, hematocrit, electrocardiography, chest radiography (posteroanterior view), transthoracic echocardiography, cardiac catheterization (if performed), and computed tomography angiography (CTA) for congenital heart disease.

Intraoperative variables included cardiopulmonary bypass (CPB) time, laterality of BDG (unilateral or bilateral), whether BDG was primary or followed prior neonatal palliation, and any concomitant procedures. Postoperative data included systemic oxygen saturation, Glenn pressures, duration of mechanical ventilation, inotropic support, intensive care unit (ICU) stay, total hospital stay, and early mortality. Follow-up assessments included oxygen saturation, symptom improvement, cardiac catheterization findings (if available), reintervention rates, and time to FO.

The criteria used to determine the need for a fenestrated Fontan included: mean PA pressure >15 mm Hg, transpulmonary gradient >8 mm Hg, ventricular end-diastolic pressure >12 mm Hg, or evidence of elevated pulmonary vascular resistance on cardiac catheterization.

Statistical analysis

Data analysis was carried out with IBM SPSS Statistics for Windows, Version 24 (Released 2016; IBM Corp., Armonk, New York, United States) or Stata Statistical Software: Release 14 (College Station, TX: Stata Corp LP). Categorical variables were compared with Fisher’s exact or Pearson’s chi-square tests. Continuous data were shown as mean ± standard deviation or median (minimum, maximum), as required based on data distribution, which was determined by the Shapiro-Wilk test. Predictors of mortality and other outcomes were assessed by Cox proportional hazards or logistic regression models, with all preoperative, intraoperative, and postoperative variables included as covariates. Missing data were handled using listwise deletion, as the proportion of missing values was <5% for all variables. A p-value <0.05 was regarded as statistically significant.

## Results

Preoperative characteristics

One hundred seventy-three patients aged ≤12 months underwent BDG between January 2011 and December 2020. Of these, 27 were six months or younger (Group A), and 146 patients were seven to 12 months old (Group B). Both groups showed a male predominance. Preoperative features are shown in Table 1.

**Table 1:** Preoperative features BSA: body surface area; SpO_2_: oxygen saturation Continuous variables are mean ± SD and compared with Welch’s two-sample t-test; categorical variables are n (%) and compared with Pearson’s x² or Fisher’s exact test when any expected cell <5 (two-sided α=0.05). Test statistics (e.g., t(df), x²(df)) and p-values are reported. (P<0.05 is taken as significant). *Calculated using the Kolmogorov–Smirnov test for expressing continuous variables.

Parameter	Group A (Age ≤ 6 Months)	Group B (Age 7 to 12 Months)	Test Statistics	P-value*
Total (%), n=173	27 (15.6%)	146 (84.4%)		0.0001
Male, n (%)	19 (70.3%)	101 (69.2%)	χ² (1)=0.015	0.001
Female, n (%)	8 (29.7%)	45 (30.8%)		
Mean Weight (kg)	5.47 ± 1.13	7.62 ± 1.23	t (38.3)=-8.954	0.11
Mean Height (cm)	64.44 ± 5.65	73.56 ± 6.77	t (41.1)=-7.456	0.23
Mean BSA (m^2^)	0.28 ± 0.04	0.37 ± 0.04	t (36.3)=-10.740	0.19
Mean Preoperative SpO_2 _(%)	68 ± 2.7	72 ± 1.9	t (30.9)=-7.368	0.09
Preoperative Mechanical Ventilation (n)	7 (25.9%)	3 (2.1%)		0.0001
Mean Hemoglobin (g/dl)	15.77 ± 3.22	17.35 ± 2.91	t (34.3)=-2.377	0.33
Mean Hematocrit (%)	46.59 ± 9.02	52.31 ± 8.13	t (34.3)=-3.072	0.14
Mean Platelet Count (10^9^/L)	1.56 ± 0.74	1.85 ± 0.85	t (39.8)=-1.826	0.18

Ten patients required mechanical ventilatory support preoperatively, with a significantly greater proportion in Group A (seven patients) compared with Group B (three patients; p=0.0001). In Group B, nine patients had a history of a Blalock-Taussig shunt, eight had a patent ductus arteriosus (PDA), and four had previously undergone PA banding (PAB). In contrast, none of the patients in Group A had received prior surgical interventions.

All patients underwent preoperative transthoracic echocardiography. Detailed diagnoses are presented in Table 2.

**Table 2:** Diagnosis in patients undergoing BDG BDG: bidirectional Glenn shunt; DORV: double outlet right ventricle; VSD: ventricular septal defect; PS: pulmonary stenosis; RV: right ventricle; d-TGA: dextro-transposition of the great arteries; AVSD: atrioventricular septal defect; HLHS: hypoplastic left heart syndrome; ccTGA: congenitally corrected transposition of the great arteries; TAPVC: total anomalous pulmonary venous connection; NA: not applicable Frequencies are n (%). Between-group comparisons use Fisher’s exact two-sided p-values. P<0.05 is taken as statistically significant. *Calculated using the Kolmogorov–Smirnov test for expressing continuous variables. P-values were not calculated when comparators were unavailable between the two groups.

Diagnosis	Group A (Age ≤ 6 Months; n=27), n (%)	Group B (Age 7 to 12 Months; n=146), n (%)	P-value*
Tricuspid Atresia; RV Hypoplasia	14 (51.9%)	57 (39.1%)	0.12
DORV; VSD; PS	4 (14.8%)	35 (23.9%)	0.3
d-TGA; VSD; PS	5 (18.5%)	25 (17.1%)	0.09
AVSD	0	10 (6.8%)	NA
HLHS	0	9 (6.1%)	NA
Tetralogy of Fallot; Pulmonary Atresia	0	2 (1.3%)	NA
ccTGA; VSD; PS	1 (3.7%)	5 (3.4%)	0.4
Pulmonary Atresia; Intact Ventricular Septum; RV Hypoplasia	3 (11.1%)	1 (0.6%)	0.8
VSD; PS; TAPVC	0	2 (1.3%)	NA
Total	27	146	NA

CTA was performed in 133 patients (76.9%), with findings outlined in Table 3.

**Table 3:** CTA findings CTA: computed tomography angiography; SVC: superior vena cava; IVC: inferior vena cava; MAPCAs: major aortopulmonary collateral arteries; NA: not applicable Frequencies are n (%). Between-group comparisons use Fisher’s exact two-sided p-values. P<0.05 is taken as statistically significant. *Calculated using the Kolmogorov–Smirnov test for expressing continuous variables. P-values were not calculated when comparators were unavailable between the two groups.

Findings	Group A (Age ≤ 6 Months), n (%)	Group B (Age 7 to 12 Months), n (%)	P-value*
Bilateral SVC	3 (11.1%)	30 (20.5%)	0.15
Interrupted IVC	0	2 (1.3%)	NA
MAPCAs	2 (7.4%)	42 (28.7%)	0.08
Veno-Venous Collaterals	0	2 (1.3%)	NA
Right Isomerism	0	11 (7.5%)	NA
Left Isomerism	0	2 (1.3%)	NA

PA and descending thoracic aorta (DTA) anatomy and measurements are shown in Table 4.

**Table 4:** Branch PAs and DTA measurements PA: pulmonary artery; LPA: left pulmonary artery; RPA: right pulmonary artery; DTA: descending thoracic aorta; SD: standard deviation Measurements are mean ± SD. Groups are compared with Welch’s two-sample t-test after normality assessment; t(df), p-values are provided. P<0.05 is taken as statistically significant. *Calculated using the Kolmogorov–Smirnov test for expressing continuous variables. **Statistically significant differences.

PA	Group A (Age ≤ 6 Months), mean ± SD	Group B (Age 7 to 12 Months), mean ± SD	Test Statistics	P-value*
Mean Ostial LPA	5.17 ± 1.75 mm	6.68 ± 1.78 mm	t(36.7)=-4.108	0.02**
Mean Hilar LPA	4.71 ± 1.25 mm	7.05 ± 2.24 mm	t(62.1)=-7.705	0.001**
Mean Ostial RPA	6.41 ± 1.41 mm	6.95 ± 1.69 mm	t(41.1)=-1.769	0.16
Mean Hilar RPA	5.96 ± 1.71 mm	6.89 ± 1.82 mm	t(37.7)=-2.570	0.31
DTA	7.96 ± 2.01 mm	7.31 ± 1.97 mm	t(35.9)=1.548	0.29

Patients in Group A had significantly smaller mean ostial left PA (LPA) diameters (p=0.02) and mean hilar LPA diameters (p=0.001) compared with Group B. When normalized to body surface area, the differences in ostial and hilar LPA diameters between groups remained statistically significant. The diameters of the ostial or hilar right PA, main PA, or DTA did not differ significantly between the groups. Conventional angiography was performed in 11 patients, none in Group A.

Operative characteristics

Unilateral BDG was performed in 138 patients (79.8%). Bilateral BDG was performed in 33 patients with bilateral superior vena cavae, and two patients were subjected to the Kawashima procedure. Antegrade pulmonary blood flow was interrupted in 32 patients (18.5%). Concomitant interventions are reflected in Table 5.

**Table 5:** Concomitant interventions AVV: atrioventricular valve; LPA: left pulmonary artery; TAPVC: total anomalous pulmonary venous connection; PA: pulmonary artery; PPI: permanent pacemaker implantation; RPA: right pulmonary artery; LSVC: left superior vena cava; AGFI: antegrade pulmonary blood flow interruption; VSD: ventricular septal defect; NA: not applicable Frequencies are n (%). Between-group comparisons use Fisher’s exact two-sided p-values. P<0.05 is taken as statistically significant. *Calculated using the Kolmogorov–Smirnov test for expressing continuous variables. P-values were not calculated when comparators were unavailable between the two groups.

Concomitant Procedure	Group A (Age ≤ 6 Months), n (%)	Group B (Age 7 to 12 Months), n (%)	P-value*
Atrial Septectomy	5 (18.5%)	30 (20.5%)	0.11
LPA Plasty	3 (11.1%)	14 (9.6%)	0.32
TAPVC Repair	1 (3%)	3 (2.1%)	0.2
Glenn Revision to Fixed PA Band	2 (7.4%)	1 (0.6%)	0.17
RPA and LPA Thrombectomy	0	4 (2.7%)	NA
Left AVV Repair	0	3 (2.1%)	NA
Epicardial PPI	0	1 (0.6%)	NA
VSD Enlargement	0	1 (0.6%)	NA
LSVC Ligation	0	2 (1.3%)	NA
AGFI	5 (18.1%)	27 (18.4%)	0.14

CPB time and aortic cross-clamp times did not show significant differences between the two groups.

Postoperative outcomes

Postoperative outcome data are demonstrated in Table 6.

**Table 6:** Postoperative characteristics ICU: intensive care unit; IQR: interquartile range; SD: standard deviation; SpO_2_: oxygen saturation; CI: confidence interval Normal variables are represented as mean ± SD and compared with the Welch’s t-test. Time-based / skewed variables are reported as medians (IQR) and compared with the Mann–Whitney U test (reported as z, U where applicable). Two-sided α=0.05. P<0.05 is taken as statistically significant. *Calculated using the Kolmogorov–Smirnov test for expressing continuous variables.

Parameters			Group A (Age ≤ 6 Months)	Group B (Age 7 to 12 Months)	Test Statistics	P-value*
Postoperative SpO_2_ in ICU	Mean % ± SD		84.07 ± 8.98	87.09 ± 5.47	t (29.7)=-1.690	0.12
	Median % (IQR) with 95% CI		85 (84, 90)	87 (84, 90)		
Glenn Pressure in ICU	Mean mmHg ± SD		16.5 ±4.34	14.5 ± 3.68	t (33.3)=2.250	0.013
	Median mmHg (IQR) with 95% CI		15 (13, 22)	14 (12, 17)		
Maximum Inotrope Score	Mean ± SD		15.45 ± 2.3	7.73 ± 1.7	t(31.5)=16.622	0.001
	Median (IQR) with 95% CI		15 (10, 24)	6.95 (5, 10)		
Maximum Vasoactive Inotrope Score	Mean ± SD		18.73 ± 1.54	9.51 ± 0.78	t (28.5)=30.397	0.001
	Median (IQR) with 95% CI		18.42 (10, 38)	8 (5, 12)		
Postoperative Hours on Inotropes (hours), median (IQR) with 95% CI			28 (18, 44)	26 (18,61)	Mann–Whitney z≈0.09, U≈1993	0.926
Time to Extubation (hours), median (IQR) with 95% CI			6.5 (6, 12)	4.5 (4.25,9)	Mann–Whitney z≈2.97, U≈2681	0.003
ICU Stay (hours), median (IQR) with 95% CI			68 (44, 168)	63 (47,86)	Mann–Whitney z ≈1.11, U ≈2236	0.267
Hospital Stay (days), median (IQR) with 95% CI			7 (6, 8)	5.5 (5,8)	Mann–Whitney z≈2.35, U≈2532	0.019
SpO_2_ at Discharge		Mean % ± SD	80.12 ± 2.11	84.51 ± 1.98	t (35.0)=-10.025	0.085
		Median % (IQR) with 95% CI	80 (78,82)	84 (80.5, 88)		
Mortality, n (%)			5 (18.52%)	7 (4.79%)		0.01

Patients in Group A had significantly higher mean Glenn pressures, maximum inotropic scores, maximum vasoactive inotropic score (VIS), prolonged time to extubation, longer hospital stays, and higher mortality compared with Group B. The inotropic score, or VIS, was determined with the following formula: VIS = dopamine (µg/kg/min) + dobutamine (µg/kg/min) + 100 × epinephrine (µg/kg/min) + 100 × norepinephrine (µg/kg/min) + 10 × milrinone (µg/kg/min) + 10,000 × vasopressin (units/kg/min) + 50 × levosimendan (µg/kg/min) [11]. These differences represent associations and should not be interpreted as evidence that younger age independently causes higher Glenn pressures or greater inotropic requirements.

Mortality

Group A accounted for 18.52% mortality (5/27), Group B for 4.79% mortality (7/146), with Group A contributing 41.7% of all deaths in the cohort, a statistically significant difference (p=0.01). All five patients in Group A who died were on mechanical ventilation preoperatively due to respiratory distress, cyanotic spells, or seizures. Three deaths in this group were caused by severe sepsis and multiorgan dysfunction syndrome; two resulted from intractable cardiogenic shock.

In Group B, causes of death included intractable ventricular tachycardia (two patients), pulmonary hemorrhage (two patients), and ventilator-associated pneumonia with sepsis (three patients). No late deaths occurred in either group.

Morbidity

Postoperative complications were observed in 37 patients (22.9%) and included pleural effusion, pneumonia, stroke, atrioventricular block, sternal instability, chylothorax, and tracheostomy. Morbidity data are detailed in Table 7.

**Table 7:** Morbidity characteristics LRTI: lower respiratory tract infection; AV: atrioventricular; SSI: surgical site infection; BDG: bidirectional Glenn shunt; PA: pulmonary artery; NA: not applicable Frequencies are n (%). Between-group comparisons use Fisher’s exact two-sided p-values. P<0.05 is taken as statistically significant. *Determined with Fisher’s exact test. P-values were not determined when comparators were unavailable between the two groups.

Morbidity		Group A (Age ≤ 6 Months), n (%)	Group B (Age 7 to 12 Months), n (%)	P-value*
Pleural Effusion		3 (11.1%)	8 (5.4%)	0.21
LRTI/Ventilator-Associated Pneumonia		3 (11.1%)	4 (2.73%)	0.08
Tracheostomy		2 (7.4%)	4 (2.73%)	0.1
Seizure, Stroke		0	2 (1.3%)	NA
AV Block		0	3 (2.1%)	NA
Junctional Ectopic Tachycardia		0	2 (1.3%)	NA
Sternal Dehiscence, SSI		0	2 (1.3%)	NA
Chylothorax		1 (3.7%)	3 (2.1%)	0.09
Redo Surgeries	Re-exploration for bleeding	2 (7.4%)	2 (1.3%)	0.12
	Diaphragmatic plication	1 (3.7%)	5 (3.4%)	0.3
	Thoracic duct ligation	0	3 (2.1%)	NA
	Revision of BDG to the PA band	0	1 (0.6%)	NA
	Pericardial augmentation of BDG	0	1 (0.6%)	NA

Reoperations were required in 15 patients (8.67%) for issues such as bleeding, diaphragmatic plication, thoracic duct ligation, revision of BDG to PAB, and pericardial patch augmentation of the BDG. In Group B, three patients developed atrioventricular block requiring permanent pacemaker implantation, and two developed junctional ectopic tachycardia managed with medications. No rhythm disturbances were reported in Group A.

Follow-up

All patients showed symptomatic improvement during follow-up. Follow-up data are summarized in Table 8.

**Table 8:** Follow-up parameters IQR: interquartile range; CI: confidence interval; PA: pulmonary artery; SpO_2_: oxygen saturation; NA: not applicable The data has been represented as N, %, Mean ± SD x². Categorical outcomes are n (%) and compared with Fisher’s exact test. Continuous measures are mean ± SD and compared with Welch’s t-test; test statistics and p-values are reported (two-sided α=0.05). P<0.05 is taken as statistically significant. *Determined with Pearson’s chi-square test. P-values were not determined when comparators were unavailable between the two groups.

Parameter		Group A (Age ≤ 6 Months)	Group B (Age 7 to 12 Months)	Test Statistics	P-value*
Cardiac Catheterization Study Before Fontan Completion, n (%)		17 (62%)	80 (54.7%)		0.21
Fontan Performed, n (%)		12 (44.4%)	65 (44.5%)		0.91
Fenestrated Fontan, n (%)		0	11 (7.5%)		NA
Time to Completion Fontan, Years	Mean ± SD	5.5 ± 1.4	7.1 ± 0.8	t (29.2)=-5.767	0.02
	Median (IQR) with 95% CI	5.5 (5, 6.5)	7 (5.4, 7.8)		
SpO_2_ at Fontan Completion, %	Mean ± SD	81.09 ± 1.73	83.34 ± 2.1	t (41.5)=-5.991	0.085
	Median (IQR) with 95% CI	81 (77, 84)	83 (78.5, 86)		
PA Pressure at Cardiac Catheterization Before Fontan Completion, mmHg	Mean ± SD	7.33 ± 6.03	10.85 ± 3.25	t (28.9)=-2.955	0.0001
	Median (IQR) with 95% CI	7.5 (7, 11)	11.3 (9.5, 16.5)		
Type of Fontan, n (%)	Lateral Tunnel	2 (7.4%)	9 (6.2%)		0.32
	Extra-cardiac	10 (37%)	56 (38.3%)		0.29

Twenty-four patients (31%) had coil embolization of major aortopulmonary collateral arteries (MAPCAs) in the cardiac catheterization laboratory before undergoing FO; all were older than six months at the time of the procedure. Fenestration was performed when mean PA pressure exceeded 15 mmHg, transpulmonary gradient exceeded 8 mmHg, ventricular end-diastolic pressure exceeded 12 mmHg, or catheterization findings suggested elevated pulmonary vascular resistance.

Of the 161 patients discharged after BDG, 77 underwent FO following cardiac catheterization. The remaining 84 patients were under evaluation for FO. Among those who underwent FO, 11 patients received a fenestrated FO due to borderline PA pressures. The types of Fontan procedure performed did not differ significantly between the two groups.

Systemic oxygen saturation at the time of FO was 81% in Group A and 83% in the opposite group. Mean PA pressure before FO was significantly lower in the former (7.33 ± 6.03 mmHg) compared to the latter (10.85 ± 3.25 mmHg; p=0.0001).

Table 9 shows the outcomes of univariate and multivariate analyses of mortality. Age, preoperative mechanical ventilation, LPA diameter, maximum inotropic score, and maximum VIS were associated with mortality on univariate analysis. However, on multivariate analysis, only preoperative mechanical ventilation, maximum inotropic score, and maximum VIS remained independent prognostic factors of mortality. Age and LPA diameter were not indicative of it in the multivariate model. Only preoperative mechanical ventilation, maximum inotropic score, and VIS remained significant after adjustment, indicating that severity of illness rather than age per se was the dominant determinant of risk.

**Table 9:** Univariate and multivariate analysis for mortality MAPCAs: major aortopulmonary collateral arteries; LPA: left pulmonary artery; RPA: right pulmonary artery; ICU: intensive care unit; MVAS: maximum vasoactive inotropic score; MIS: median inotropic score; SpO_2_: oxygen saturation; OR: odds ratio; NA: not applicable The data has been represented as N, %, Mean ± SD x². P<0.05 is taken as statistically significant. Logistic regression results are presented as odds ratios (OR) with 95% confidence intervals, Wald z, and two-sided p-values. *Calculated using the Cox proportional hazards or logistic regression models with all preoperative, intraoperative, and postoperative variables included as potential predictors. P-values were not calculated when comparators were unavailable between the two groups.

Variables	Univariate Analysis		Multivariate Analysis		
	OR (95% CI)	P-value	OR (95% CI)	P-value*	Test statistics
Age	3.726 (1.847-10.171)	0.045	3.592 (1.695-9.876)	0.061	2.84
Gender	2.004 (0.236-6.734)	0.11	NA	NA	NA
Preoperative Ventilatory Support	4.514(1.993-16.469)	0.001	4.429 (1.784-15.973)	0.001	2.66
Preoperative SpO_2_	0.988 (0.938-1.040)	0.62	NA	NA	NA
MAPCAs	2.107 (0.692-6.395)	0.13	NA	NA	NA
LPA Size	4.102 (1.671-16.145)	0.039	3.897 (1.543-14.784)	0.07	2.36
RPA Size	0.867 (0.268-2.579)	0.32	NA	NA	NA
Postoperative SpO_2_	1.378 (0.438-4.642)	0.29	NA	NA	NA
Glenn Pressure	2.436 (0.734-6.841)	0.17	NA	NA	NA
MIS	3.592 (1.695-9.876)	0.01	3.538 (1.507-9.691)	0.03	2.66
MVAS	4.019 (1.661-15.963)	0.002	3.971 (1.607-15.841)	0.001	2.36
Time to Extubation	1.634 (0.574-4.884)	0.23	NA	NA	NA
ICU Stay	2.215 (0.723-7.315)	0.18	NA	NA	NA
Hospital Stay	1.986 (0.527-6.261)	0.09	NA	NA	NA

## Discussion

The FO represents the definitive palliative strategy for patients with a functionally UVH. Fontan physiology places the systemic and pulmonary circulations in series, supported by a single functional ventricle [12]. Initial neonatal palliation aims to establish unobstructed systemic outflow and systemic and pulmonary venous return, along with controlled pulmonary blood flow. In patients with UVH and no pulmonary outflow obstruction, pulmonary blood flow may increase excessively as pulmonary vascular resistance decreases during the first week of life. These patients often require PAB to limit pulmonary overcirculation and reduce PA pressure. Conversely, patients with obstructed pulmonary outflow may undergo a systemic-to-pulmonary shunt or BDG before eventual FO [13].

The BDG has been performed for over six decades and is well established as a safe and effective palliative procedure in UVH patients with low pulmonary vascular resistance and PA pressures [8,14,15]. It increases systemic oxygen saturation and reduces volume overload on the systemic ventricle [16,17], and it serves as an intermediate step toward FO.

BDG outcomes are favorable in older children, with low operative mortality consistently reported in the literature [4,18-21]. However, only a limited number of studies from Western centers have explored BDG outcomes in infants. This study analyzed outcomes in 173 patients who underwent BDG at age one or younger, including 27 infants aged six months or younger. Early mortality was 6.93% overall, with five of the 12 deaths occurring in the younger group. Mortality was significantly higher in infants aged six months or younger, likely due to greater clinical severity, as evidenced by a higher proportion of patients in Group A requiring preoperative mechanical ventilation and having higher maximum inotropic scores and VIS (p=0.001). On multivariate analysis, preoperative mechanical ventilation, rather than younger age, was independently associated with increased mortality.

These outcomes are consistent with a review by Chang et al. [10], which included 17 infants aged 4.2 to 6.6 months and reported one perioperative death. Albanese et al. [21] reported outcomes of BDG in 27 patients aged under two years (mean 14.2 months), with four perioperative deaths (15%). Their analysis attributed these deaths to complex cardiac anatomy, severe atrioventricular valve regurgitation (AVVR), or pulmonary venous obstruction, rather than patient age alone.

In our cohort of patients aged six months or younger, morbidity occurred in nine patients (33.3%), and three patients (11.1%) required reoperation. Two underwent re-exploration for bleeding, and one underwent diaphragmatic plication for phrenic nerve palsy. Similarly, Kogon et al. [22] reported 27% morbidity in a series of 270 infants aged younger than six months, though most complications were non-life-threatening and resulted only in prolonged ICU and hospital stays. Freedom et al. [23] emphasized that appropriate preoperative selection is key to reducing mortality and morbidity.

In Western populations, hypoplastic left heart syndrome (HLHS) is the most frequent UVH subtype, followed by tricuspid atresia [24-26]. In contrast, tricuspid atresia is the predominant diagnosis in Indian cohorts. In a previous study from our institution involving 215 patients, tricuspid atresia accounted for 40% of cases, while double outlet right ventricle (DORV) with a ventricular septal defect (VSD) that is remote from the aorta and pulmonary stenosis (PS) accounted for 36% [27]. In our research, tricuspid atresia was observed in 41% of patients, followed by DORV with VSD and PS (22.5%), dextro-transposition of the great arteries (d-TGA) with VSD and PS (17.3%), and HLHS (5.2%). Thus, while the dominant ventricle is typically the right ventricle in Western patients, it is often smaller or hypoplastic in Indian patients due to the high prevalence of tricuspid atresia.

AVVR is known to worsen outcomes in patients with UVH physiology [28]. In our study, three patients (2.1%) aged over six months had AVVR, comparable to the incidence reported by Friedman et al. [28]. This observation may suggest that AVVR is more likely to develop with age [29]. All three patients underwent atrioventricular valve repair, although existing studies suggest that such interventions are associated with poor survival [30]. However, the sample size in our study is too small to make definitive conclusions.

Among our patients, 33 (19%) had bilateral SVCs. In two cases, the left SVC was hypoplastic and was ligated; the remaining patients underwent bilateral BDG as the initial stage of palliation. Two patients with interrupted inferior vena cava had the Kawashima procedure as the definitive palliation. Ando et al. [30] reported that bilateral SVCs did not increase surgical risk and had no impact on central PA growth or reintervention rates following bilateral BDG. In contrast, Iyer et al. [31] found that bilateral BDG was associated with increased thrombosis, higher reintervention rates, increased mortality, and a lower rate of FO. In our experience, we did not observe any of these complications in patients who underwent bilateral BDG.

In our study, 24 patients (31%) had coil embolization of MAPCAs in the cardiac catheterization laboratory prior to FO. MAPCAs can increase volume loading on the systemic ventricle, elevate systemic venous and PA pressures, and contribute to prolonged pleural effusions and hemodynamic instability [32]. These effects may render patients unsuitable for the FO [33]. Therefore, careful preoperative screening and occlusion of MAPCAs are essential to optimize outcomes in single-ventricle palliation.

At our institution, interruption of antegrade pulmonary blood flow (APBF) during BDG is guided by a standardized protocol [27]. Following completion of the BDG anastomosis, SVC pressure is monitored using a cannula positioned percutaneously in the internal jugular vein. If SVC pressure is <15 mmHg and the pressure tracing is non-pulsatile, APBF is typically preserved. If SVC pressure is elevated or pulsatile, temporary occlusion of the main PA is performed. If this results in a fall in SVC pressure >3 mmHg and/or a non-pulsatile tracing, APBF is interrupted. In this study, APBF was interrupted in 32 patients (Group A: n=5; Group B: n=27).

Mainwaring et al. [18] reported improved survival in patients with interrupted APBF, noting that preserved APBF was indicative of higher risks of chylothorax, pleural effusions, prolonged hospitalization, and anastomotic site aneurysms. Another study involving 43 patients compared outcomes in those with and without preserved APBF. Patients with preserved APBF (n=21) had greater postoperative oxygen saturation rates but also greater central venous pressure and a greater risk of chylothorax [20,34].

In our cohort, all systemic-to-pulmonary shunts and PDA were occluded before BDG to prevent volume overload. Most MAPCAs were occluded prior to FO. At our center, preserving APBF is sometimes preferred to delaying FO, particularly in stable patients. However, for those in whom APBF was interrupted during BDG, FO is typically scheduled within two to three years to prevent the occurrence of pulmonary arteriovenous malformations [27].

Concomitant procedures were frequently performed during BDG, including atrial septectomy in 20.2% of patients (Group A: n=5; Group B: n=30), left PA plasty in 9.8% (Group A: n=3; Group B: n=14), and total anomalous pulmonary venous connection repair in 2.3% (Group A: n=1; Group B: n=3). These procedures are well supported in the literature and have been associated with improved outcomes when performed in conjunction with BDG [21,26,27].

Patients aged six months or younger had significantly higher Glenn pressures, maximum inotropic scores, VISs, extubation times, and hospital stays. However, the duration of inotropic support and ICU stay did not differ significantly between groups. These findings align with those of prior studies [4,27,35]. Postoperative systemic oxygen saturation improved in both groups at discharge and during follow-up, consistent with previously reported outcomes [4,19].

All patients demonstrated symptomatic improvement following BDG. Cardiac catheterization was performed during follow-up in patients with progressive cyanosis, declining oxygen saturation, or reduced exercise tolerance. Overall, 47% of patients (Group A: n=12; Group B: n=65) underwent FO, with the remainder under evaluation. The mean interval from BDG to FO was 6.77 ± 1.1 years, which is longer than reported in Western studies [7,8,20]. This delay may be attributed to two primary factors. First, patients with uninterrupted APBF often experience symptom relief and may delay follow-up. Second, limited institutional resources and the need to prioritize urgent repairs for complex congenital heart defects may postpone elective FO. Additionally, delayed presentation was noted in 81.5% of patients (Group A: n=22; Group B: n=119).

A fenestrated FO was performed in 14% of patients (Group A: n=0; Group B: n=11) due to elevated PA pressures identified during catheterization. In high-risk patients, fenestration has been proven to improve surgical outcomes and reduce hospital stay [36].

Pulmonary arteriovenous malformation (PAVM) formation is an important interstage concern after BDG due to the absence of hepatic venous effluent to the pulmonary circulation. Reported incidence ranges from 70-100% in Glenn physiology. Moore et al. first established this association and demonstrated the role of hepatic factors in preventing PAVMs [37]. Kavarana et al. further clarified the clinical relevance and the need to restore hepatic flow to both lungs to reverse or prevent progression [38]. These considerations highlight the importance of vigilant interstage monitoring and timely FO.

Limitations 

Despite the insights gained, there are some notable limitations. First, its retrospective nature introduces the potential for information bias, as data were collected from existing records rather than through a prospective, standardized protocol. Second, although this was conducted at a high-volume tertiary care center, it remains single-center, which may restrict the generalizability of the outcomes to other institutions with different patient populations, resources, or surgical expertise. Third, the unequal sample sizes between the age groups, particularly the smaller cohort of patients aged six months or younger, reduce the statistical power for subgroup analyses. Fourth, selection bias may have occurred if sicker or more complex patients were preferentially selected for earlier intervention, although all eligible cases within the specified time frame were included. Fifth, the heterogeneity of underlying cardiac anatomies introduces multiple confounding variables, making it difficult to isolate the effect of age alone on outcomes. Finally, the length and consistency of follow-up varied among patients, which may lead to incomplete capture of late morbidity and mortality. Future prospective multicenter studies with standardized protocols and longer, more uniform follow-up periods could help validate these findings and clarify optimal timing for BDG in younger patients.

## Conclusions

This study was designed to compare outcomes in patients who underwent BDG at six months or younger versus those aged seven months to one year, evaluate the interval to FO, and identify early and late outcomes predictors in an Indian cohort. Our analysis revealed significantly higher early mortality in infants aged six months or younger, driven primarily by the clinical severity of illness and increased requirement for preoperative mechanical ventilation rather than by younger age alone. Nevertheless, early BDG in infants offered the potential benefits of unloading the systemic ventricle sooner and reducing the number of preceding palliative procedures. At follow-up, these younger patients also demonstrated reduced PA distortion, suggesting that timely intervention can promote more favorable vascular remodeling.

These findings have important clinical implications for centers managing UVH. By highlighting risk factors such as the need for mechanical ventilation and high inotropic scores, our study results stress the importance of careful patient selection, perioperative management, and comprehensive follow-up. The study also addresses knowledge gaps in the Indian context, where tricuspid atresia rather than HLHS is the predominant UVH subtype and where resource constraints and delayed patient presentation can extend the interval to FO. Our findings should inform clinical decision-making regarding the timing of BDG in infants, particularly in resource-limited settings. Understanding the risks and benefits of earlier versus later BDG can help clinicians personalize treatment strategies, potentially improving survival and reducing morbidity among infants with UVH. Future research, especially prospective and multicenter trials, could refine these insights further and contribute to evidence-based guidelines on the optimal timing for BDG and subsequent FO.

## References

[1] Kaulitz R, Hofbeck M (2005). Current treatment and prognosis in children with functionally univentricular hearts. Arch Dis Child.

[2] Pennington DG, Nouri S, Ho J (1981). Glenn shunt: long-term results and current role in congenital heart operations. Ann Thorac Surg.

[3] Reddy VM, Liddicoat JR, Hanley FL (1995). Primary bidirectional superior cavopulmonary shunt in infants between 1 and 4 months of age. Ann Thorac Surg.

[4] Bridges ND, Jonas RA, Mayer JE, Flanagan MF, Keane JF, Castaneda AR (1990). Bidirectional cavopulmonary anastomosis as interim palliation for high-risk Fontan candidates. Early results. Circulation.

[5] Pridjian AK, Mendelsohn AM, Lupinetti FM (1993). Usefulness of the bidirectional Glenn procedure as staged reconstruction for the functional single ventricle. Am J Cardiol.

[6] di Carlo D, Williams WG, Freedom RM, Trusler GA, Rowe RD (1982). The role of cava-pulmonary (Glenn) anastomosis in the palliative treatment of congenital heart disease. J Thorac Cardiovasc Surg.

[7] Kopf GS, Laks H, Stansel HC (1990). Thirty-year follow-up of superior vena cava-pulmonary artery (Glenn) shunts. J Thorac Cardiovasc Surg.

[8] Glenn WW (1989). Superior vena cava-pulmonary artery shunt. Ann Thorac Surg.

[9] Reddy VM, McElhinney DB, Moore P (1997). Outcomes after bidirectional cavopulmonary shunt in infants less than 6 months old. J Am Coll Cardiol.

[10] Chang AC, Hanley FL, Wernovsky G (1993). Early bidirectional cavopulmonary shunt in young infants. Postoperative course and early results. Circulation.

[11] Wernovsky G, Wypij D, Jonas RA (1995). Postoperative course and hemodynamic profile after the arterial switch operation in neonates and infants. A comparison of low-flow cardiopulmonary bypass and circulatory arrest. Circulation.

[12] Fontan F, Baudet E (1971). Surgical repair of tricuspid atresia. Thorax.

[13] Khairy P, Poirier N, Mercier LA (2007). Univentricular heart. Circulation.

[14] Castaneda AR (1992). From Glenn to Fontan. A continuing evolution. Circulation.

[15] Trusler GA, Williams WG, Cohen AJ (1990). William Glenn lecture. The cavopulmonary shunt. Evolution of a concept. Circulation.

[16] Lamberti JJ, Spicer RL, Waldman JD (1990). The bidirectional cavopulmonary shunt. J Thorac Cardiovasc Surg.

[17] Hopkins RA, Armstrong BE, Serwer GA, Peterson RJ, Oldham HN Jr (1985). Physiological rationale for a bidirectional cavopulmonary shunt. A versatile complement to the Fontan principle. J Thorac Cardiovasc Surg.

[18] Mainwaring RD, Lamberti JJ, Moore JW (1996). The bidirectional Glenn and Fontan procedures—integrated management of the patient with a functionally single ventricle. Cardiology in the Young.

[19] Hawkins JA, Shaddy RE, Day RW (1993). Mid-term results after bidirectional cavopulmonary shunts. Ann Thorac Surg.

[20] Salmon AP, Sethia B, Silove ED (1989). Cavopulmonary anastomosis as long-term palliation for patients with tricuspid atresia. Eur J Cardiothorac Surg.

[21] Albanese SB, Carotti A, Di Donato RM (1992). Bidirectional cavopulmonary anastomosis in patients under two years of age. J Thorac Cardiovasc Surg.

[22] Kogon BE, Plattner C, Leong T, Simsic J, Kirshbom PM, Kanter KR (2008). The bidirectional Glenn operation: a risk factor analysis for morbidity and mortality. J Thorac Cardiovasc Surg.

[23] Freedom RM, Nykanen D, Benson LN (1998). The physiology of the bidirectional cavopulmonary connection. Ann Thorac Surg.

[24] Hoffman JI, Kaplan S (2002). The incidence of congenital heart disease. J Am Coll Cardiol.

[25] Rao PS (2000). Tricuspid atresia. Curr Treat Options Cardiovasc Med.

[26] Talwar S, Sandup T, Gupta S (2018). Factors determining early outcomes after the bidirectional superior cavopulmonary anastomosis. Indian J Thorac Cardiovasc Surg.

[27] Nelson DP, Schwartz SM, Chang AC (2004). Neonatal physiology of the functionally univentricular heart. Cardiol Young.

[28] Friedman KG, Salvin JW, Wypij D (2011). Risk factors for failed staged palliation after bidirectional Glenn in infants who have undergone stage one palliation. Eur J Cardiothorac Surg.

[29] Imai Y, Takanashi Y, Hoshino S (1997). Modified Fontan procedure in ninety-nine cases of atrioventricular valve regurgitation. J Thorac Cardiovasc Surg.

[30] Ando Y, Fukae K, Hirayama K, Oe M, Iwai T (2014). Impact of bilateral superior venae cavae on outcome of staged Fontan procedure. Ann Thorac Surg.

[31] Iyer GK, Van Arsdell GS, Dicke FP (2000). Are bilateral superior vena cavae a risk factor for single ventricle palliation?. Ann Thorac Surg.

[32] Spicer RL, Uzark KC, Moore JW, Mainwaring RD, Lamberti JJ (1996). Aortopulmonary collateral vessels and prolonged pleural effusions after modified Fontan procedures. Am Heart J.

[33] Kurotobi S, Sano T, Kogaki S (2001). Bidirectional cavopulmonary shunt with right ventricular outflow patency: the impact of pulsatility on pulmonary endothelial function. J Thorac Cardiovasc Surg.

[34] Gaies MG, Gurney JG, Yen AH (2010). Vasoactive-inotropic score as a predictor of morbidity and mortality in infants after cardiopulmonary bypass. Pediatr Crit Care Med.

[35] Zhang T, Shi Y, Wu K, Hua Z, Li S, Hu S, Zhang H (2016). Uncontrolled antegrade pulmonary blood flow and delayed Fontan completion after the bidirectional Glenn procedure: real-world outcomes in China. Ann Thorac Surg.

[36] Lemler MS, Scott WA, Leonard SR, Stromberg D, Ramaciotti C (2002). Fenestration improves clinical outcome of the Fontan procedure: a prospective, randomized study. Circulation.

[37] Moore JW, Kirby WC, Madden WA, Gaither NS (1989). Development of pulmonary arteriovenous malformations after modified Fontan operations. J Thorac Cardiovasc Surg.

[38] Kavarana MN, Jones JA, Stroud RE, Bradley SM, Ikonomidis JS, Mukherjee R (2014). Pulmonary arteriovenous malformations after the superior cavopulmonary shunt: mechanisms and clinical implications. Expert Rev Cardiovasc Ther.

